# Comparison of Intravenous Ampicillin–sulbactam Plus Nebulized Colistin with Intravenous Colistin Plus Nebulized Colistin in Treatment of Ventilator Associated Pneumonia Caused by Multi Drug Resistant *Acinetobacter Baumannii:* Randomized Open Label Trial

**DOI:** 10.22037/ijpr.2019.112466.13775

**Published:** 2019

**Authors:** Elham Pourheidar, Mehrdad Haghighi, Mehran Kouchek, Mir Mohammad Miri, Seyedpouzhia Shojaei, Sara Salarian, Rezvan Hassanpour, Mohammad Sistanizad

**Affiliations:** a *Department of Clinical Pharmacy, School of Pharmacy, Shahid Beheshti University of Medical Sciences, Tehran, Iran. *; b *Department of Infectious Diseases, Imam Hossein Teaching and Educational Center, Shahid Beheshti University of Medical Sciences, Tehran, Iran. *; c *Department of Critical Care Medicine, Imam Hossein Medical and Educational Center, Shahid Beheshti University of Medical Sciences, Tehran, Iran.*

**Keywords:** Acinetobacter, Acute kidney injury, Colistin, Nebulizer, Pneumonia, Ventilator-associated

## Abstract

The purpose of this study was evaluating the efficacy and safety of intravenous (IV) ampicillin–sulbactam plus nebulized colistin in the treatment of Ventilator-Associated Pneumonia (VAP) caused by MDR *Acinetobacter *(MDRA) in ICU patients as an alternative to IV plus nebulized colistin. In this single-blinded RCT, one group received IV colistin and another group IV ampicillin–sulbactam (16 and 12 patients from total 28 patients, respectively) for 14 days or since clinical response. Both groups received nebulized colistin by mesh nebulizer. There were no statistically significant differences between the 2 groups in baseline characteristics and previous antibiotic therapy. In follow up period, no significant difference was observed between 2 groups in rate of microbiological eradication, clinical signs of VAP improvement, survival rate and length of hospital as well as ICU stays. Although we have found no significant differences in Acute Kidney Injury (AKI) incidence between two groups, comparison of cumulative patient-days with stages 2 and 3 AKI with days with no or stage 1 AKI, according to AKIN criteria, revealed significant difference in IV colistin versus IV ampicillin–sulbactam group (*p* = 0.013). The results demonstrated that the high dose IV ampicillin–sulbactam plus nebulized colistin regimen has comparable efficacy with IV plus nebulized colistin in the treatment of VAP caused by MDRA, with sensitivity to colistin only, with probably lower incidence of kidney injury.

## Introduction

Hospital-Acquired Pneumonia (HAP) including Ventilator-Associated Pneumonia (VAP), the most common infection in the Intensive Care Unit (ICU), is associated with prolonged hospital and ICU stay, high costs and poor outcomes (1). The incidence of VAP has been reported around 30% among mechanically ventilated patients with a mortality rate between 27 to 76%, depending on the organism. Pneumonia due to *Pseudomonas* and *Acinetobacter* is associated with a higher mortality rate (2, 3). 

*Acinetobacter Baumannii* is one of the most common Gram-negative pathogens in VAP, especially late VAP that occurs after 5 to 7 days of admission to the hospital. It also accounts for more than 36% of HAP cases in Asia (4-7). Many studies have been done to find the optimal treatment for VAP caused by MDR pathogens (8, 9). 

An antibiotic needs a concentration of more than 10 to 25 times the Minimal Inhibitory Concentration (MIC) to be effective against bacteria in the pulmonary purulent secretion. With most antibiotics like colistin, this level of concentration in ELF and pulmonary secretions cannot be reached with intravenous administration alone (10, 11). A major reason for the superiority of combination therapy with intravenous and nebulizer compare with intravenous therapy alone can be drug delivery directly to the Epithelial Lining Fluid (ELF), supplying acceptable drug concentration at the site of infection, and overcoming drug resistance in patients with pneumonia by using nebulizer devices along with reduced systemic absorption and side effects (12). Systemic adverse effects of colistin like renal toxicity which is the most common side effect with reported incidence of 19 to 54 percent in various studies, it depends on its serum concentration. Due to insignificant systemic absorption of inhaled colistin, this incidence in patients receive nebulized colistin is less than subjects receive intravenous form (13-16). 

This study was designed to evaluate the efficacy and safety of intravenous ampicillin–sulbactam plus nebulized colistin in the treatment of VAP caused by MDR *Acinetobacter Baumannii *with sensitivity to colistin only in ICU patients as an alternative to intravenous plus nebulized colistin.

## Experimental

*Patients and setting*

This open label Randomized Clinical Trial (RCT) was conducted at a 30-bed medical-surgical intensive care unit of Imam Hossein medical center, affiliated to Shahid Beheshti University of Medical Science (SBMU) in Tehran, Iran. This study has been approved by institutional review boards of ethics committee of SBMU (IR.SBMU.PHARMACY.REC.1397.007) and has been registered in Iranian registry of clinical trials, too (IRCT20120703010178N18).

Inclusion criteria were defined as confirmed VAP based on clinical and radiological signs and positive sputum culture of MDR *Acinetobacter Baumannii* with sensitivity to colistin or colistin and ampicillin–sulbactam only, which is defined by disk diffusion in our setting, with concentration > 10^5^ CFU/mL in patients who mechanically ventilated for >48 h (17). Written consent was obtained from patients or their families. 

The patient was excluded if he had pneumonia before intubation, history of moderate or severe hypersensitivity reactions to beta-lactam antibiotics or colistin, dialysis, history of receiving appropriate antibiotics for this episode of VAP for more than 96 h before recruitment, co-infection with another organ(s), Acute Respiratory Distress Syndrome (ARDS), chest trauma with fracture of the sternum, ribs, or both, immunosuppression including patients with active cancer, exacerbation of chronic bronchitis within the last 30 days, tuberculosis on treatment, suspected atypical pneumonia, cystic fibrosis, pregnancy, and lactation.

*Study protocol and assessments*

The patients were allocated in intravenous (IV) colistin or intravenous high dose ampicillin–sulbactam group of the study using block randomization. The patients in the IV colistin group received 9 × 10^9^ units loading dose followed by the 4.5 × 10^9^ units twice daily colistin (Ronak darou, Iran). Subjects in IV ampicillin–sulbactam group received continuous infusion of high dose ampicillin–sulbacatam (Dana Pharmaceutical, Iran) 24 g daily (6 g (at a ratio 2:1) four times a day, each dose infused over 6 h). Both groups received nebulized colistin 2 × 10^9 ^units every 8 h with mesh nebulizer (Solo Nebulizer device which is designed for mechanically ventilated patients, Aerogen, USA). The dose and dosing interval for systemic agents was adjusted according to the serum creatinine levels and creatinine clearances. 

Prolong infusion, which is defined as continuous infusion or extended infusion over 3 or 4 h, revealed a better antibacterial effect compared to short-term infusion in several studies (18, 19). Ampicillin–sulbactam IV solution is stable in Normal Saline (NS) just for 8 h and it could not be used for 24 h infusion period. To solve this problem, we administered ampicillin-sulbactam as 6 g (4:2) every 6 h and each dose was infused over 6 h. 

The following variables were recorded for every patient enrolled in this study: age, sex, ICU diagnosis on admission based on ICD10 codes and Acute Physiologic and Chronic Health Evaluation (APACHE) II score on the recruitment day; maximum temperature, leukocyte count (WBC) and serum creatinine level daily and Procalcitonin (PCT) level, chest X-ray and sputum culture on baseline, and then 3^rd^, 7^th^, 10^th^ and 14^th^ days after recruitment to the study. Follow up period for study was considered 14 days. In the case of clinical response or exclusion before the 14^th^ day of the study, the mentioned data were documented until that day. Also, ICU and the Hospital Length of Stay (ICULOS and HLOS), duration of mechanical ventilation before and after recruitment to the study, and 28 days and total mortality of ICU and hospital were recorded.

The primary outcome was a microbiological eradication. As secondary outcomes, we evaluated clinical cure; renal toxicity (based on AKIN criteria), the mortality rate during 28 days, and ICU and hospital length of stay.

Clinical response was defined as resolution of pneumonia related to signs and symptoms, including fever and bronchial secretions, for at least 48 h, Presence of one of the following signs considered as clinical failure: fever (T ≥ 38 °C) or hypothermia (T < 35.5 °C), copious and purulent pulmonary secretion, more than 50% increase in pulmonary infiltrate on CXR, lack of recovery in P_a_O2/ F_i_O2, septic shock or multi-organ failure (20, 21). In case of clinical failure or superinfection with pathogens other than *Acinetobacter Baumannii* in following sputum cultures. So the patient was excluded and the treatment was changed according to the physician’s perception.

*Sample size*

The sample size of the study was calculated with G power tool (version 3.1.9.2, university Kiel, Germany) using test for two proportions function, considering type I error of 0.05, power of 0.8, the proportion of expected treatment effect of 45% for colistin and 75% for ampicillin–sulbactam in treatment of VAP due to MDR *Acinetobacter*. The number of participants calculated 14 in each group.

*Statistical analysis*

 All statistical analyses were performed using SPSS for Windows (Version 21.0; SPSS Inc., Chicago, IL, USA). All data was compared using per-protocol analysis. Categorical variables were compared using χ2 test or Fisher exact test, as appropriate. Continuous variables were tested for normality of distributions by Kolmogorov–Smirnov test, and then compared by Student’s *t*-test or the Mann-Whitney U test, as appropriate. All the tests were two-tailed, and a *P*-value of < 0.05 was considered significant.

## Results

**Figure 1:**
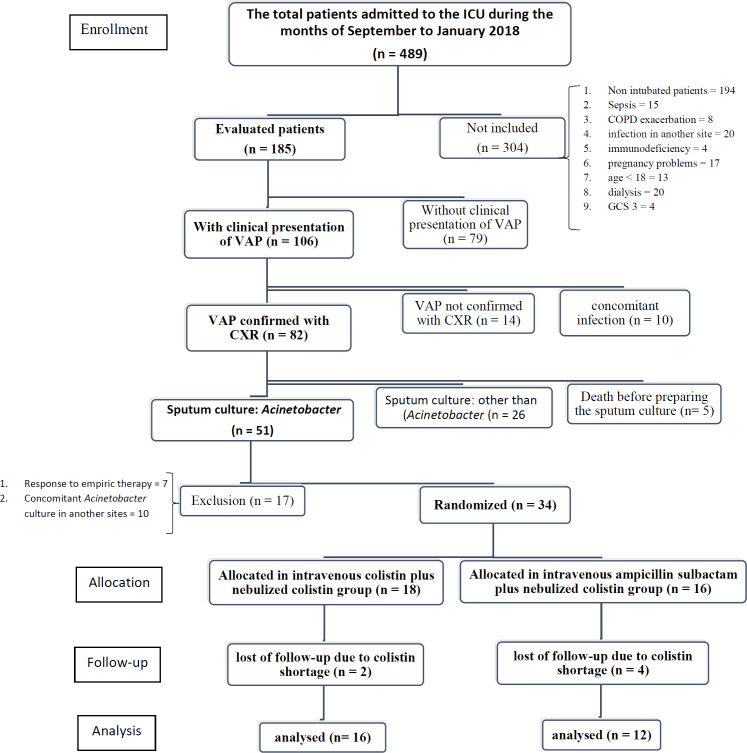
Disposition of patients with MDR *Acinetobacter* VAP included in the analysis of the impact of intravenous high dose ampicillin-sulbactam plus nebulized colistin and intravenous plus nebulized colistin. VAP: ventilator associated pneumonia; COPD: Chronic obstructive pulmonary disease; GCS: Glasgow Coma Scale; CXR: Chest X Ray

Four-hundred eighty-nine patients were admitted to the ICU of Imam Hossein Medical Center from September to January 2018. Fifty-one patients diagnosed with VAP based on clinical and radiological signs plus sputum culture of MDR *Acinetobacter Baumannii *with sensitivity to colistin or colistin and ampicillin–sulbactam only. Twenty-three of them excluded because of response to empiric therapy (n = 7), concomitant infection with MDR *Acinetobacter Baumannii (A. Baumannii)* in other sites (n = 10) and errors in administration of medications due to shortage of colistin (4 and 2 subjects in ampicillin–sulbactam and colistin arms, respectively). From the remaining 28 patients, 16 patients were allocated in IV colistin group (12 sputum culture of MDR *Acinetobacter Baumannii *with sensitivity to colistin only and 4 with sensitivity to colistin and ampicillin–sulbactam only) and 12 patients in IV ampicillin–sulbactam group (12 sputum culture of MDR *Acinetobacter Baumannii *with sensitivity to colistin only and 4 with sensitivity to colistin and ampicillin–sulbactam only). Data are revealed in Figure 1. There were no statistically significant differences in baseline characteristics (Table 1) and empiric antibiotics which were started by intensivist or infectious disease specialist before inclusion to the study (Table 2) between the 2 groups. 

**Figure 2:**
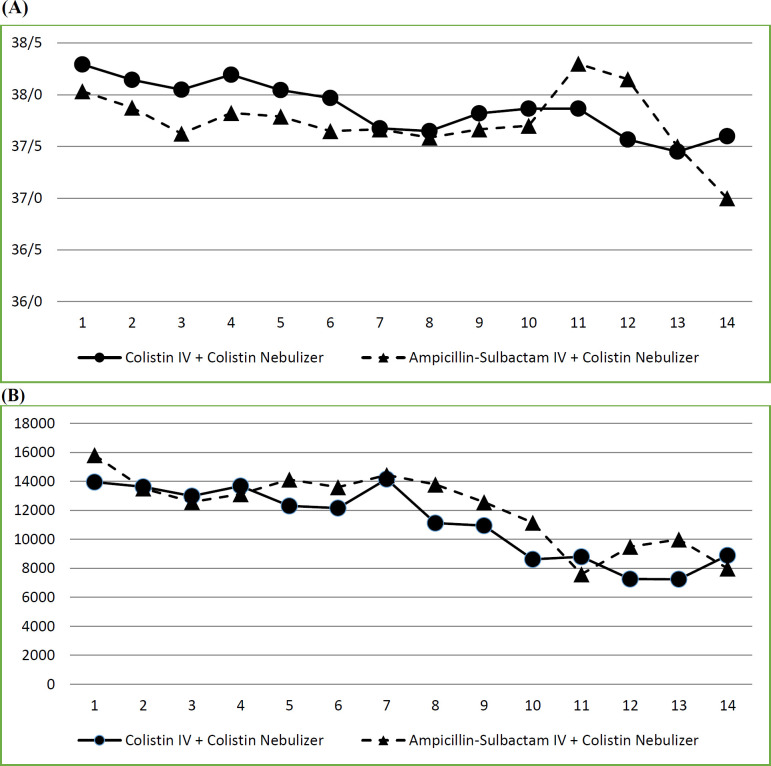
Interventions effect on clinical signs of VAP (comparison of A: Temperature, B: Leukocyte count in intervention and control arms of the study).

Patients were monitored daily for clinical signs of VAP including WBC and temperature. No significant difference was observed between the WBC of patients in two arms of the study during the follow-up period. Regarding the maximum temperature of the patients in two groups, the difference was non-significant, except in the 3^rd^ day of the study that the mean of temperature in IV ampicillin–sulbactam group was significantly lower than IV colistin group (37.6 ± 0.4 against 38.1 ± 0.6; *p* = 0.037). Data are shown in Figure 2.

About PCT, we have found no significant differences in PCT levels of baseline in recruitment day and 3^rd^, 7^th^, 10^th^, and 14^th^ days of the study between 2 groups. Also, differences among PCT levels at mentioned days with baseline did not show statistically significant differences between the two arms of the study. More than 80% reduction in PCT, compared to the baseline level, was observed just in one patient in IV colistin group on 3^rd^ and 7^th^ days of the study and in 2 patients (one in IV colistin group and one in IV ampicillin–sulbactam group) on 14^th^ day of the treatment. 

Clinical cure with successful discontinuation of antibiotics happened in 5 (31.2%) and 4 (33.3%) subjects, in colistin and ampicillin-sulbactam arms, respectively which 2 of isolated MDR *Acinetobacter Baumannii* species in both groups were sensitive to ampicillin–sulbactam. Mean treatment duration was 8 ± 3 (range, 3 to 14 days) and 9 ± 3 days (range, 4 to 14 days) in colistin and ampicillin–sulbactam groups, respectively (*p* = 0.562).

As shown in the Table 3, surveillance culture on the 3^rd^ day of the study showed microbiological eradication in one subject in colistin arm and 4 cases in ampicillin–sulbactam group. This was 1 and 2 on the 7^th^ day of the study. Just in one patient in ampicillin–sulbactam group microbiological eradication was documented on 10^th^ day of the study. 

Microbiologic Response definition: Eradication: elimination of the original causative organism(s) from the same site during or upon completion of therapy; Persistence: failure to eradicate the original causative organism(s) from sites previously listed, whether or not signs or inflammation are present; Superinfection: development of a new lower respiratory tract during treatment or within 3 days after treatment has been completed that is due to a new or resistant pathogen not recognized as the original causative organism(s); Reinfection: elimination of the initial infecting pathogen followed by its replacement with a new species or a new serotype or biotype of the same organism in the presence of signs or symptoms of infection after completion of therapy; Colonization: development of a positive sputum culture that yields a bacterial strain other than the primary causative isolate that appears >48 h after initiation of therapy, persists in at least two repeated cultures, and is not associated with fever, leukocytosis, persistence or progression of pneumonia, or evidence of infection at a distant site; Indeterminate: circumstances in which it is not possible to categorize the microbiologic response because of death and the lack of opportunity to perform further cultures, the withdrawal of the subject from the study before follow-up cultures can be obtained, incomplete microbiologic data, or concurrent treatment of the patient with a potentially effective anti-infective agent that is not part of the study protocol. The name of the agent and the dose and duration of this therapy must be recorded. The duration of therapy will affect decisions about patient evaluability and outcome.

**Figure 3:**
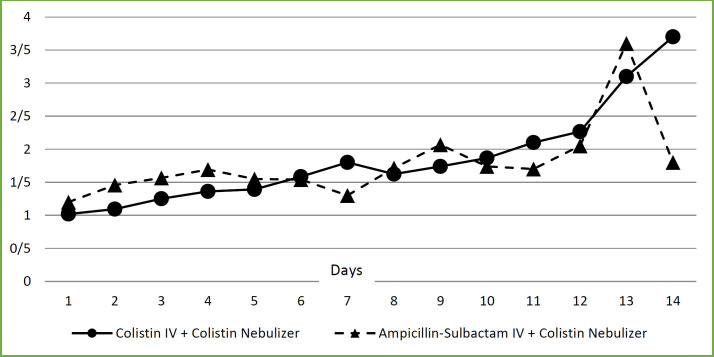
Interventions effect on serum creatinine

Although there were no significant differences between daily creatinine levels of the patients, and number of patients who suffered from AKI in two arms of the study (8/16 in IV colistin versus 4/12 in IV ampicillin–sulbactam groups; *p* = 0.378), Figure 3, comparison of cumulative days with stages 2 and 3 AKI with days with no or stage 1 AKI, according to AKIN criteria, in two arms of the study revealed significantly higher number of patient-days in IV colistin versus IV ampicillin–sulbactam arm (19/110 versus 5/88, respectively; *p* = 0.013) (Table 4).

As presented in Table 5, ICU intubation period, ICU-LOS, and hospital-LOS were not significantly different in IV ampicillin–sulbactam group compared to IV colistin arm. About 28-day mortality 2 (16.7%) and 6 (37.5%) patients expired in ampicillin–sulbactam and colistin groups, respectively. Although this was not statistically significant (*p* = 0.227), clinically it could be important as percent of expired subjects in the IV colistin group were more than twice compared with IV ampicillin–sulbactam arm of the study.

**Table 1:** Baseline characteristics

			**Intervention groups**						**sig** ^a,b,c^
			**Colistin IV + Colistin Nebulizer**			**Ampicillin-Sulbactam IV + Colistin Nebulizer**			
			**count**	**Mean ** ± ** SD** ^f^	**Min–Max**	**count**	**Mean ** ± ** SD**	**Min–Max**	
Sex	Female		6			6			0.508^a^
	Male		10			6			
Age				60 ±19	22–87		59 ± 16	32–85	0.859^b^
Weight				79 ± 20	55–120		82 ± 10	68–98	0.589^b^
Lean Body Weight				53.50 ± 8.98	34.58–70.98		54.48 ± 9.02	43.41–70.24	0.779^b^
ICD10Code^d^	T		5			2			0.613^a^
	C		2			1			
	G		5			5			
	J		1			1			
	I		1			2			
	S		2			0			
	K		0			1			
APACHEII				15 ± 4	9–20		16 ± 5	12–31	0.709^c^
ampicillin-sulbacatam sensitvity^e^		S	4			3			1.00^a^
		R	12			9			
PCT base			16	6.06 ± 18.46		11	1.76 ± 3.07		0.505^c^
intubation period (before recruitment)				16 ± 12	5–47		13 ± 4	7–20	0.339^a^

^a^Chi-square; ^b^Independent samples *t*-test; ^c^Mann Whitney; ^d^ICD10 code definition: T = Injuries to unspecified part of trunk, limb or body region, C = Malignant neoplasms, G = Diseases of the nervous system (loss of consciousness, ICH), J = Diseases of the respiratory system, I = Diseases of the circulatory system (cerebrovascular accident, pericardial effusion), S = Injuries to specified part of body, K = Diseases of the digestive system; APACHE II, acute physiologic and chronic health evaluation; ^e^ampicillin-sulbacatam sensitivity definition: S = Sensitive, R = Intermediate or Resistant; ^f^Standard Deviation

**Table 2:** Previous antibiotics which patient received empirically before recruitment

		**Intervention groups**		**Sig** ^a^
		**Colistin IV + Colistin Nebulizer**	**Ampicillin-Sulbactam IV + Colistin Nebulizer**	
Previous Antibiotic 1	None	1	0	0.543
	Meropenem	8	8	
	Imipenem	2	1	
	Piperacillin-Tazobactam	2	3	
	Ceftazidime	2	0	
	Cefepime	1	0	
Previous Antibiotic 2	None	1	0	0.452
	Levofloxacin	1	0	
	Vancomycin	13	11	
	Clindamycin	0	1	
	Teicoplanin	1	0	
Previous Antibiotic 3	None	11	8	0.631
	Ciprofloxacin	4	4	
	Amikacin	1	0	

^a^Chi-square.

**Table 3:** Microbiologic Response

		**Intervention groups**		**sig** ^a^
		**Colistin IV + Colistin Nebulizer**	**Ampicillin-Sulbactam IV + Colistin Nebulizer**	
3^rd^ day	Eradication	1	4	0.347
	Persistence	6	2	
	Superinfectin	4	3	
	Colonization	2	2	
	Indeterminate	3	1	
3^rd^ day (second pathogen)	Superinfectin	4	1	.
7^th^ day	Eradication	1	2	0.370
	Persistence		1	
	Reinfection		1	
	Colonization	2	2	
	Indeterminate	2		
10^th^ day	Eradication		1	
	Persistence	1		
10^th^ day (second pathogen)	Colonization	1		.

^a^Chi-square.

**Table 4:** Cumulative days with or without AKI

		**AKI**		**Sig** ^a^
		**No** ^b^ ** or stage 1** ^c^	**Stage 2** ^c^ ** or 3** ^c^	
Intervention groups	IV colistin + neb colistin	91	19	0.013
	IV ampicillin + neb colistin	83	5	

^a^Chi-square; ^b^No AKI; ^c^AKIN stages: stage 1 = increase in SCr ≥ 0.3 mg/dL; stage 2 = increase in SCr ≥ 2.0 × baseline;

stage 3 = increase in SCr ≥ 3.0 × baseline or SCr ≥ 4.0 mg/dL (with acute rise of ≥0.5 mg/dL) or initiation of RRT (Renal replacement therapy).

**Table 5:** ICU intubation period, hospital length of stay and ICU length of stay

	**Colistin IV + Colistin Nebulizer**		**Ampicillin-Sulbactam IV + Colistin Nebulizer**		
	**Mean ** ± ** SD**	**Min–Max**	**Mean ** ± ** SD**	**Min–Max**	**sig** ^a^
ICU Intubation period after recruitment	18 ± 10	3–41	21 ± 19	4–63	0.623^a^
Adjusted^c^ ICU Intubation period after recruitment	17 ± 8	3–28	15 ± 10	4–28	0.761^a^
Total HLS	44 ± 14	23–67	55 ± 23	13–100	0.165^a^
Adjusted^c^ Total HLS	27 ±2	23–28	27 ± 4	13–28	0.795^b^
HLS after recruitment	25 ± 12	7–46	38 ± 18	4–64	0.053^a^
Adjusted^c^ HLS after recruitment	21 ± 8	7–28	25 ± 7	4–28	0.188^b^
Total ICULS	36 ± 12	14–62	33 ± 22	11–80	0.696^a^
Adjusted^c^ Total ICULS	26 ± 4	14–28	22 ± 7	11–28	0.093^b^
ICULS after recruitment	20 ± 10	3–41	21 ± 18	4–63	0.967^a^
Adjusted^c^ ICULS after recruitment	19 ± 8	3–28	16 ± 10	4–28	0.405^a^

^a^Independent samples T-test; ^b^Mann Whitney; ^c^In adjusted analyze group the maximum evaluation time is 28 days; HLS: hospital length

of stay; ICULS: ICU length of stay.

## Discussion

Due to the increased prevalence of resistant *Acinetobacter* species, many studies have been conducted to evaluate the effectiveness of different antibiotic regimens (13). *Acinetobacter* defined as Multi-Drug Resistant (MDR) if it became resistant to at least one agent in three or more of five following effective antimicrobial categories: cephalosporins, carbapenems, ampicillin-sulbactam, fluoroquinolones, and aminoglycosides (22, 23).

The first consensus reported by the committee of 10 Asian countries in 2019, about treatment of hospital pneumonia in Asian countries, recommends the use of high doses sulbactam as an alternative for the treatment of *A. Baumannii* infections (24). Data comparing ampicillin–sulbactam with colistin in the treatment of MDR *A. Baumannii* is few. In a systemic review and meta-analysis about treatment of pneumonia due to MDR *Acinetobacter Baumannii*, clinical response and survival of high dose sulbactam and combined intravenous and nebulized colistin were significantly superior to intravenous colistin alone (with clinical cure rate of 72.7% and 81.8% respectively *vs.* 45.5% for IV colistin alone) (9). As sulbactam alone is not available in all countries, the ampicillin–sulbactam combination has been used as an alternative for sulbactam, with efficacy similar to colistin in the treatment of VAP caused by MDR *Acinetobacter Baumannii* (with clinical improvement rate of 13.3% *vs.* 15.3%, respectively) (25, 26). Our study set out with the aim of assessing the efficacy and safety of intravenous ampicillin–sulbactam plus nebulized colistin in the treatment of ventilator-associated pneumonia caused by MDR *Acinetobacter Baumannii *with sensitivity to colistin or colistin and ampicillin–sulbactam only, in ICU patients as an alternative to intravenous plus nebulized colistin. Our important finding was similar efficacy of two regimens and the most interesting finding was the lower nephrotoxicity rate in ampicillin–sulbactam based regimen. In accordance with the present result, previous study by Betrosian AP and colleagues in 2008 showed similar efficacy of high dose ampicillin–sulbactam (9 g every 8 h) compared with colistin (3 million units every 8 h) in the treatment of VAP caused by MDR *Acinetobacter Baumannii *resistant to ampicillin–sulbactam and sensitive to colistin. These clinical results are consistent with the pharmacokinetic findings that indicate better pulmonary penetration of ampicillin and sulbactam in comparison with colistin. These studies indicated that administration of intravenous ampicillin–sulbactam provided adequate concentration in lung tissue (alveolar fluid to serum concentration ratio greater than 50%) and could be a good choice for lower respiratory tract bacterial infections (20, 27 and 28). We used ampicillin–sulbactam 24 g daily as continuous infusion plus nebulized colistin. In a study by Betrosian *et al.*, ampicillin–sulbactam regimens with 27 and 36 g daily doses showed comparable clinical efficacy which also were similar with colistin in the treatment of VAP produced by ampicillin–sulbactam resistant and colistin sensitive MDR *Acinetobacter Baumannii* (26). Khalili H *et al.* also reported comparable clinical and microbiological response of meropenem/colistin combination and meropenem/ampicillin-sulbactam combination in treatment of VAP due to carbapenem-resistant *A. baumanni* (29). 

Despite a higher number of patients with nephrotoxicity in the IV colistin group (50% *versus* 33.3% IV ampicillin–sulbactam group), this difference was not statistically significant between two arms of our study. This could be due to the small sample size of the current study. Also, we compared cumulative days with stages 2 and 3, according to AKIN criteria, with days with no or stage 1 AKI in two arms of the study which revealed significantly higher number of patient-days in IV colistin group versus IV ampicillin–sulbactam arm (17.3% versus 5.7%, respectively; *p* = 0.013). In accordance with our findings, the Betrosian study also reported a lower rate of kidney injury in ampicillin–sulbactam group than colistin (15.4% *vs.* 33.3% in ampicillin-sulbactam and colistin groups, respectively) (25). Mosaed and his colleague also reported lower incidence of nephrotoxicity with ampicillin–sulbacatam versus colistin (8% *vs.* 54% in ampicillin-sulbactam and colistin groups, respectively) (14).

In one arm, we used a combination of nebulized colistin with systemic colistin, as recommended in IDSA, 2016 guideline (17). In the treatment of pneumonia, antibiotic concentration in the ELF plays an important role in clinical response (30). Beta-lactams, aminoglycosides, and vancomycin have little penetration into the ELF, and the concentration generated in ELF is often less than 50% of their serum concentration (31). Also about colistin, in several studies, it has been documented that this agent could not reach to the MIC of MDR Gram-negative bacteria like *Acinetobacter* and *Pseudomonas* in the ELF (20, 32-34). Increasing systemic dose of antibiotics to reach therapeutic levels in ELF will increase the probability of systemic adverse events. In order to overcome mentioned problems, using nebulized antibiotics has been recommended which deliver drugs directly into ELF and could produce concentrations greater than 100 times the MIC of most bacteria in airways, including MDR organisms, without causing systemic toxicity (35). The IDSA guideline 2016 recommends the use of inhaled antibiotics with low penetration to ELF including aminoglycosides and polymyxins in the treatment of VAP (17).

In another arm of the study, combination of nebulized colistin with systemic ampicillin–sulbactam has been used. Different outcomes in different studies have been defined for this combination. An *in-vitro* study which evaluated the efficacy of ampicillin-sulbactam plus colistin on the ampicillin–sulbactam resistant MDR *A. Baumannii* suggests that although this combination therapy has a high synergistic effect *in-vitro*, this effect is more likely to be observed against species are resistant to colistin (14 have synergistic effects among 21 colistin-resistant strains), while among the 12 species susceptible to colistin, the combination of ampicillin–sulbactam and colistin was mainly associated with antagonistic effects (9 cases) (36). While a clinical study of 39 ICU patients with VAP caused by Carbapenem-Resistant *Acinetobacter Baumannii* (CRAB) which sensitive to colistin and ampicillin–sulbactam showed that co-administration of high dose ampicillin–sulbactam and colistin compare to colistin alone resulted significantly higher incidence of clinical cure (the initial clinical response rate, defined as the clinical response within the first 4 to 5 days of treatment, was 70% in the combined group, 15.8% in the single treatment group (*p* = 0.001)). In this study, colistin was used at a dose of 3 million units 3 times a day and ampicillin–sulbactam was given at a dose of 6 g 4 times a day. So, the results of this study did not confirm the antagonistic effect of a combination of ampicillin–sulbactam with colistin in colistin sensitive *A. Baumanni* species in clinical settings (20). Also, in another study, Kalin and Pongpech confirmed the synergistic effect of colistin and sulbactam combination against *A. Baumannii*. In these studies, the addition of sulbactam to colistin reduced MIC of both antibiotics against *A. Baumannii* (37, 38). In a letter to editor Kempf *et al.*, concluded that although the optimum treatment is not currently well established for MDR *A. Baumannii* infections, the combination of colistin with sulbactam may provide significant benefit over monotherapy and improve the chance of survival (39).

## Conclusion

In conclusion, the results demonstrated that the high dose ampicillin–sulbactam plus nebulized colistin regimen has comparable efficacy with intravenous plus nebulized colistin in treatment of VAP caused by MDR *Acinetobacter Baumannii *with sensitivity to colistin only, with probably lower incidence of kidney injury, based on AKIN criteria, and could be considered as an alternative treatment.

## Limitations of Our Study

Shortage of colistin and mesh nebulizer were our main limitation in this study.
